# Can the digital economy development curb carbon emissions? Evidence from China

**DOI:** 10.3389/fpsyg.2022.938918

**Published:** 2022-09-02

**Authors:** Xiaoli Hao, Shufang Wen, Yuhong Li, Yuping Xu, Yan Xue

**Affiliations:** ^1^School of Economics and Management, Xinjiang University, Urumqi, China; ^2^Beibu Gulf Ocean Development Research Center, Beibu Gulf University, Qinzhou, China; ^3^School of Economics and Finance, Xi’an Jiaotong University, Xi’an, China; ^4^Center for Innovation Management Research, Xinjiang University, Urumqi, China; ^5^School of Economics and Trade, Hunan University, Changsha, China

**Keywords:** digital economy, carbon emissions, spatial spillover effect, double threshold, inverted N-type

## Abstract

“Carbon neutrality, carbon peaking” is China’s national commitment to the whole world about its plans to manage global climate change. China faces many severe challenges in fulfilling its commitments to reduce emissions. China’s digital economy is currently booming, and whether it can provide opportunities for reducing regional carbon emissions is worth exploring. This study constructed a comprehensive system to evaluate the development of its digital economy based on China’s regional data and empirically tested the direct, indirect, and spatial effects of the comprehensive development of digital economy on regional carbon emissions. In addition, it examined the special stage characteristics using a Hansen threshold model. This study found the following: first, the digital economy significantly suppresses carbon emissions in general, notably with a spatial spillover effect to neighboring provinces. Secondly, an analysis of the mechanism shows that the comprehensive development of a digital economy can restrain regional carbon emissions through industrial progress and the optimization of energy consumption. Third, there are double thresholds, special driving trends and an “inverted N-type” relationship with development. Fourth, a spatial heterogeneity analysis revealed that significant “local” and “neighboring” impacts on the reduction of carbon emissions only exist in the central and eastern areas. This study has a reference value for releasing the dividend of digital economy development and reducing carbon emissions.

## Introduction

The increasingly serious problem of global warming is one of the current global challenges for humans, and the international community has a consensus to take effective policy measures to actively respond to climate change and accelerate the transition to a low-carbon society (Doren et al., 2020). According to the data in the “BP World Energy Statistics Yearbook,” China’s carbon emissions accounted for approximately 30% of global emissions in 2020, and the carbon emission per unit of GDP was 6.7 tons of carbon dioxide per 10,000 USD, which was 1.5- and 1.8-fold those of the world average, respectively. As a country that is a major consumer of energy and source of carbon emissions, how to break the “strange circle” of environmental and economic growth and realize the attractive vision of sustainable and high-quality development is China’s rigid task and prescribed action (Li et al., 2019). China proposed “carbon peaking and carbon neutrality” in the 75th session of the United Nations General Assembly – a solemn commitment that embodies China’s determination to manage climate change and also highlights China’s image as a responsible major country for increasing its green policies after the epidemic and building a community with a shared future for mankind (Wang et al., 2019).

Simultaneously, digital information technology has become increasingly integrated into all fields of economic and social development and is currently a vital factor in the globally competitive landscape by enabling quality, efficiency, and power. Investing in the digital economy is a strategic move toward capitalizing on the new opportunities presented by the new round of technological revolutions and industrial transformation (Bhattacharya et al., 2015). The latest research from the Chinese Academy of Information and Communications Technology (Haidian, China) indicates that China’s digital economy contributed 38.6% of its GDP in 2020, which is still low when compared with those of developed countries. The digital economy continues to grow in size and influence and serves as a powerful catalyst for the country to improve technological innovation efficiency, drive industrial transformation, and improve product quality (Wu et al., 2021). The “Digital China” strategy is changing China’s economic development model from the pursuit of rapid economic growth to high-quality economic development, and the digital economy has received constant attention and high priority (Li et al., 2019). The environmental improvement effect of the digital economy has also become one of the primary focuses of scholarly research. The digital economy can promote the upgrading of whole industrial chain through the knowledge spillover effect (Moyer and Hughes, 2012). With the progress of information technology and its innovative application, online information has become an important driving force for the management of environmental pollution. For example, e-commerce can eke out space for the development of industries that consume large amounts of energy and emit a high level of emissions through the crowding-out effect, optimizing the industrial structure. Thus, there is an enormous potential role for digital technology in energy conservation and the reduction of emissions (Alam and Murad, 2020; Wu et al., 2021).

Therefore, can the digital economy change the extensive economic growth mode and promote China’s carbon emission reduction? If so, does this effect have phased characteristics? How does it work? Does it have spatial spillover effects? The study of these issues will not only enrich digital economy research but will also be of practical significance in implementing the new green development concept and promoting China’s “dual carbon” goal.

The structure of rest of this article is arranged as follows: section “Literature review” is a literature review. Section “Mechanistic analysis” is an analysis of mechanisms. Section “Research design” explains the primary research models and describes the data. Section “Empirical results and discussion” is the empirical results and discussions, which explores the direct effect, mediating effect, threshold and spatial spillover effects. Section “The robustness test” is a test of robustness. Section “Further analysis-regional heterogeneity test” is a detailed analysis of the test for regional heterogeneity, and the final portion summarizes the conclusions and suggests policy implications.

## Literature review

Early studies on the digital economy were primarily on the theoretical level and included such topics as its definition (Gerhard and Martin, 2005), connotation, and the digital dividend (Lundborg et al., 2012). With the rapid growth of digital economy, scholars have attempted to build the framework for a system to evaluate the digital economy (Xu and Zhang, 2020; Wang et al., 2021). Moreover, academics have focused their attention on the economic and social welfare consequences of the digital economy. Existing studies have conducted extensive and in-depth research on the digital economy at the three levels described below. On the macroeconomic level, the digital economy helps to optimize the allocation of factors and the precise matching of supply and demand (Zhou et al., 2018), which not only improves total factor productivity, but also promotes inclusive and macroeconomic growth (Bertani et al., 2020). The digital economy at the meso level is conducive to the transformation of manufacturing, its upgrading, and high-quality development (Alam et al., 2018; Chen and Zhang, 2021). Additionally, the digital economy significantly contributes to the efficiency of technological innovation on the micro level (Nambisan et al., 2019). Some scholars have studied a low-carbon transition for the digital industry by delineating the production structure factor into three components (Wang et al., 2022a).

The reduction of carbon emissions has long been the focus of academic research, and the relative results are extremely substantive. Most of the studies have examined regional differences in carbon emissions and discovered that they strongly aggregate spatially with a geographical pattern of regional imbalanced distribution (Chang et al., 2019). Studies have discovered important factors, such as technological innovation, energy intensity and structure, GDP, and industrial structure (Khan et al., 2020). Investment in research and development (Cho and Sohn, 2018; Fernández et al., 2018) also contributes to carbon emissions.

Academic research on the relationship between the digital economy and carbon emissions is relatively scarce. Studies have found that the digital economy has significantly reduced regional carbon emissions through technological progress (Wang et al., 2019). A similar situation has also been identified in the Yangtze River Delta from improving technological innovation and increasing the economic scale (Wang et al., 2022b). However, earlier studies on the digital economy and carbon emission relationship are limited and have inconsistent results (Chen, 2022). There are two opposing viewpoints. First, the use of digital technology devices and infrastructure will increase the demand for energy (Peng, 2013). Large amounts of electricity will be consumed by devices of information and computer technology (ICT) and technological industrialization (Salahuddin and Alam, 2015). Secondly, the growth of the internet and e-commerce industry could have a crowding-out effect, resulting in the elimination of energy-intensive industries (Shobande, 2021). Digital technologies can contribute to green development by disrupting the geographic boundaries, influencing energy consumption, and optimizing resource integration and environmental decision-making (Dubey et al., 2019; Ren et al., 2021). Some scholars found that the digital economy would change the renewable energy structure from two dimensions – consumption and generation – by promoting the ability of governments to govern (Shahbaz et al., 2022).

The digital economy will inevitably go through multiple stages of development, and the different development periods of digital technology could differentially impact carbon emissions and their reduction. Generally speaking, the initial application will subvert the modes of operation of traditional industries by substantially improving production efficiency and reducing energy consumption (Bhujabal et al., 2021). However, the digital economy has network effects, and large-scale investments in digital infrastructure and its utilization will increase the intensity of demands for electricity, thus, resulting in a surge in demand for rare metals and energy consumption, which could postpone economic and ecological benefits (Li et al., 2020; Hao et al., 2022). Furthermore, the factors of production tend to congregate in high-return areas, which could have a detrimental influence on regional ecological efficiency, and the ability of digital economy to reduce carbon emissions could be greatly hindered at this stage of development. With the completion of digital infrastructure construction, digital information technology will enable various industries to increase accurate production and sales and thus, improve industrial efficiency (Tang et al., 2021).

Therefore, more studies focus on the one-sided digital economy, such as the role of ICT advances on the environment, and few scholars have examined the digital economy as a whole to conduct in-depth discussions on its effects on green development. This study focuses on the impact and internal mechanism of the development of a digital economy on carbon emissions and discusses its spatial spillover effect, mediation effect and possible threshold effect based on the panel data from 2013 to 2018 in 30 provinces. These are all municipalities that are directly under the Central Government and autonomous regions in China.

The possible marginal contributions lie in this study. First, academics have utilized a variety of methodologies to quantify the absolute magnitude and relative level of the digital economy, but they have yet to develop a consensus or authoritative standard. This study measured the development of digital economy from multi-levels and multi-dimensions, including four primary indicators (digital economy foundation, digital penetration, digital industry development level, and digital economy potential) and 12 secondary indicators, which will aid in gaining a more thorough grasp of the digital economy. Secondly, this study tried to dissect the impact and its internal path from both theoretical and empirical perspectives, complementing the few relevant studies. Third, considering that only a few researchers have studied the impact in a dynamic way, this study utilized a threshold model to reveal the dynamic changes in the iterative process of digital economy. Finally, considering the possible spatial characteristic, this study extended and deepened the study of spatial spillover effect to some extent.

## Mechanistic analysis

**FIGURE 1:**
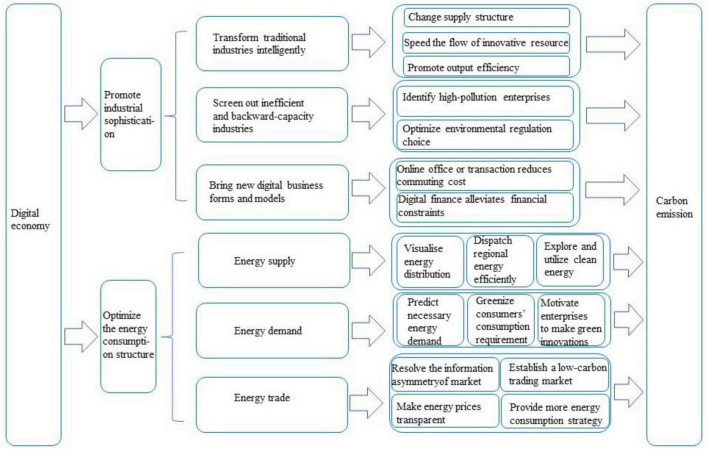
Diagram of the analysis of mechanisms.

Although existing scholarly studies have confirmed that the digital economy has a positive impact on the improvement of ecological and environmental efficiency, few studies have conducted an in-depth excavation of its mechanism of action. Digitalization plays an important role in reducing carbon emissions and shows great potential for ecological and environmental benefits. Its theoretical mechanism is shown in Figure 1.

The digital economy is progressively decarbonizing by promoting industrial sophistication. This is primarily reflected in three aspects. First, the digital economy can realize industrial digitalization by intelligently transforming traditional industries and empowering all aspects of the traditional industrial chain. To begin with, based on the advantage of rich data resources, the digital economy matches the production of products with the demands of consumers, which changes the traditional supply chain, thus, resulting in a more flexible production scale (Wang et al., 2019). This not only increases output efficiency but also reduces unnecessary losses of resources and emissions of pollutants, which are all consistent with the concept of green development (Atkinson and Mckay, 2007). In addition, it can improve the sharing of innovative knowledge of enterprises. Technological spillover enables highly efficient industries to obtain more innovative resources and accelerate the transformation of low-carbon manufacturing processes, as well as the continuous improvement of energy efficiency (Khan et al., 2020; Usman et al., 2021). Secondly, the digital economy helps to screen out inefficient and backward-capacity industries. Digital technology supports carbon footprint tracking, which optimizes the decision-making capabilities of environmental regulation. Internet technology affects the energy efficiency and consumption of enterprises by improving the technology for utilizing energy, which results in the improvement of environment (Feng et al., 2017). Third, the digital economy fosters digital industrialization and generates new digital business forms and models. In particular, digital finance improves the efficiency of allocation and timely distribution of capital elements, thereby alleviating the financing constraints of the energy industry and lowering energy consumption (Faisal et al., 2018).

By optimizing the structure of energy consumption, the digital economy can gradually achieve lower carbon emissions. On the energy supply side, digital technology enables the predictable demand of energy and increases the visibility of its distribution and efficiency of dispatching energy resources (Bhattacharya et al., 2015). The real-time monitoring of carbon emissions forces the intelligence and intensification of energy production using digital technologies. Digital technology can improve the overall energy efficiency by optimizing the consumption and absorption of new energy sources and reducing the reliance on traditional fossil fuels (Murshed, 2020). On the energy demand side, the government employs technology, such as digital twins, to determine the lowest economic cost to more effectively manage demand side carbon emissions (Lange et al., 2020). Consumers have higher requirements in energy price, stability and cleanliness. Enterprises are motivated to use digital technology to improve the manufacturing process, develop clean energy technology and develop the transition from high production that produces many pollutants that are dangerous for the environment to production that is green (Stock et al., 2018). Energy trading has become feasible owing to the resolution of the issue of information asymmetry in the market. Energy prices are transparent owing to algorithms that are assisted by technology. In addition, massive data provides market participants with more strategic alternatives to energy consumption. The advancement of digitalization promotes the establishment of a low-carbon green energy trading market, optimizes the structure of energy consumption, and assists China in achieving the goal of carbon peak and carbon neutrality.

## Research design

### Model settings

#### Construction of the basic model

To investigate the direct impact of the digital economy on the carbon intensity, the basic model (1) was established as follows:


(1) $$lnC_{i,t}=\beta_{0}+\beta_{1}DIGE_{i,t}+\beta_{2}X_{i,t}+\mu_{i}+\varepsilon_{i,t}$$

where *C*_*i,t*_ is the carbon emission intensity in the *t*-th year of the *i*-th province; *DIGE*_*i*,*t*_ is the development level of digital economy; *X*_*i,t*_ represents a series of control variables; μ_*i*_ represents the individual fixed effects of i province, and ε_*i*,*t*_ stands for the random error term.

#### Construction of the mediation model

To analyze the possible mechanism of indirect impacts based on to the analysis of mechanism described above, the advanced industrial structure and the energy consumption structure (ECS) were tested as the intermediary variables. The following mediation model (2) was constructed based on Baron and Kenny (1986):


(2) $$lnC_{i,t}=\alpha_{0}+\alpha_{1}DIGE_{i,t}+\sum control+\mu_{i}+\varepsilon_{i,t}$$

(3) $$Med_{i,t}=\beta_{0}+\beta_{1}DIGE_{i,t}+\sum control+\mu_{i}+\varepsilon_{i,t}$$

(4) $$lnC_{i,t}=\gamma_{0}+\gamma_{1}DIGE_{i,t}+\gamma_{2}Med_{i,t}+\sum control+\mu_{i}+\varepsilon_{i,t}$$

where *Med*_*i*,*t*_ refers to the intermediary variables, including industrial structure upgrading (inds) and the ECS.

#### Construction of the Hansen threshold model

Because development of the digital economy has different stages, it may not be a simple linear relationship. Therefore, it is necessary to test the possible threshold effect and its special driving trend. The threshold model (3) (Hansen, 1999) was established as follows:


(5) $$C_{i,t}=\delta_{0}+\delta_{1}DIGE_{i,t}\times I\left(DIGE_{i,t}\leq \pi \right)+\delta_{2}DIGE_{i,t}$$

$$\times I\left(DIGE_{i,t}>\pi \right)+\delta_{3}X_{i,t}+\mu_{i}+\varepsilon_{i,t}$$

where *DIGE* is the threshold variable. *I* is an indicator function, and π is the threshold value.

#### Construction of the spatial Durbin model

The spatial Durbin model can control the spatial effects with more robust estimation results (LeSage and Pace, 2009; Elhorst, 2014). Carbon emissions can also have some spatially related characteristics owing to the increasing frequency of cross-regional economic exchanges. To verify the spatial spillover effect of the digital economy on carbon emissions, the spatial interaction terms of carbon emission intensity, digital economy and control variables were introduced on the basis of model (1), and the spatial Durbin model was established as follows:


(6) $$lnC_{i,t}=\emptyset_{0}+\rho WlnC_{i,t}+\varphi_{1}WDIGE_{i,t}+\emptyset_{1}DIGE_{i,t}$$

$$+\varphi_{2}WX_{i,t}+\emptyset_{2}X_{i,t}+\mu_{i}+\varepsilon_{i,t}$$

### The variable description and evaluation index system

#### Explained variable and explanatory variable

##### Explained variable: carbon emission intensity (C)

Reducing carbon emissions is consistent with the green development trend of China’s economy, which will help to establish an innovative and long-term mechanism for the green and low-carbon transformation of China’s economy (Wang et al., 2019). The primary source of carbon emissions is the consumption of fossil fuels, such as coal, crude oil and natural gas (Wu et al., 2021). Carbon emissions were estimated based on the current international methods of calculation using the carbon emission calculation method issued by the Intergovernmental Panel on Climate Change (IPCC). Carbon intensity was characterized as the sum of products of consumption of different types of carbon-containing energy and their corresponding CO_2_ emission factors:


(7) $$C=\sum_{i=1}^{8}E_{i}\times SSC_{i}\times CEF_{i}$$

where *C* represents the estimation of carbon emissions; *E* represents the consumption of each fossil energy; *SSC* represents the decal factor of each fossil source, and *CEF* represents the carbon emission coefficient of each type of fossil fuel.

##### Core explanatory and threshold variable: Digital economy development index

The digital economy improves the efficiency by which resources are utilized and drives economic structural changes. However, scholars have not yet provided a unified standard for the connotation of the digital economy, and the current main measurement method is the scale measurement of the digital economy (Xu and Zhang, 2020) or the construction of an evaluation index system. As described by Chen (2022), an evaluation index system with four dimensions was built to evaluate it and included the digital economy foundation, digital popularization, digital industry development, and digital economy potential. The entropy value method was used as a more objective method to evaluate the index. The specific indicators are shown in Table 1.

**TABLE 1:** Index system for evaluating the development of digital economy.

Target level	Criterion level	Index level	Index attribute
Digital economy	Digital economy foundation	Fiber optic cable length/per square kilometer	+
		Number of electronic reading rooms	+
		Number of cell phones per capita	+
		Number of broadband ports per capita	+
	Digital popularization	Broadband penetration rate (%)	+
		Digital TV subscriber rate (%)	+
	Digital industry development	Total business volume of telecommunication industry (billion yuan)	+
		Added value of tertiary industry (billion yuan)	+
	Digital economy potential	Regional R&D personnel (10,000 people)	+
		Total number of R&D projects	+
		R&D intensity (%)	+
		Number of employees in the IT industry (10,000 people)	+

##### Description of core variables

**FIGURE 2:**
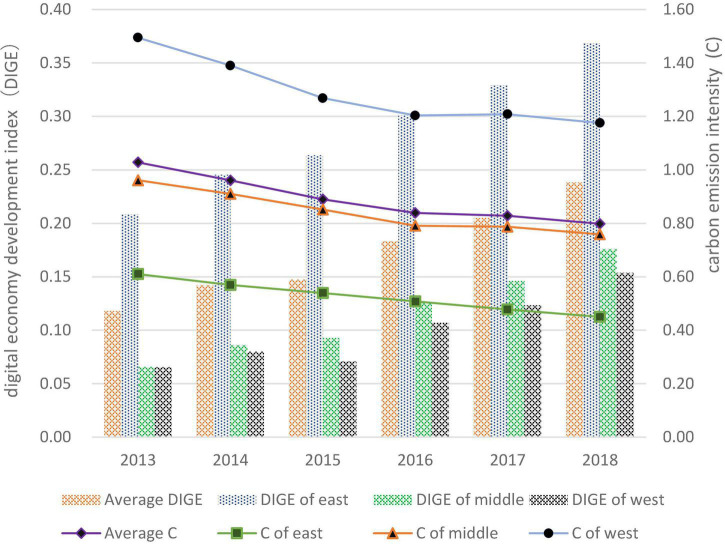
Digital economy development index and carbon emission intensity in 2013–2018.

The comprehensive level of development of the digital economy and the intensity of carbon emissions were estimated as shown in Figure 2. As shown in Figure 1, the level of development of the digital economy continued to increase in general from 2013 to 2018, and the carbon emissions showed a trend of decreasing yearly. In particular, the level of development of the digital economy is higher in the east than the national average, and although the eastern and western regions are catching up yearly, they are still below the national average. Carbon emissions in the western region are higher than the national average, while those in the central and eastern regions are slightly lower than and far below the national average, respectively.

#### Mediating variables

The digital economy can alleviate the pressure of reducing carbon emissions by upgrading the industrial structure and improving the ECS based on the theoretical mechanistic analysis described above. Therefore, the following mechanistic variables were selected:

##### Industrial structure upgrading

First, industrial digitalization empowers the traditional models of industrial production, optimizes the allocation of resources, integrates and builds a whole industrial chain that encompasses production, transportation, marketing and recycling; technological spillover effects caused by upgrading industrial structure can promote the utilization of efficient energy (Wu et al., 2020). Secondly, digital industrialization is a manifestation of the advanced industrial structure, which can accelerate the pace of industry to technology-intensive industries, which continuously induces energy saving and builds an environmentally friendly society. The GDP was divided into three parts based on the three industries by referring to the practice of defining the advancement of industrial structure (Wang et al., 2019). The added value of each part was used as a sub-vector of the spatial vector based on the proportion of the added value of each part in the GDP, which constitutes a three-dimensional vector *X*_0_ (*x*_1,0_, *x*_2,0_, *x*_3,0_). The angle between this vector and the vector of the first *X*_1_ (1, 0, 0), second *X*_2_ (0, 1, 0), and third *X*_3_ (0, 0, 1)industry was calculated, respectively.


$$\theta_{j}=arccos\frac{X_{1,j}*X_{1,0}+X_{2,j}*X_{2,0}+X_{3,j}*X_{3,0}}{\sqrt{X_{1,j}^{2}X_{2,j}^{2}X_{3,j}^{2}}\sqrt{X_{1,0}^{2}X_{2,0}^{2}X_{3,0}^{2}}}j=1,2,3$$

The formula for calculating the industrial structure advanced value of *P* is as follows:


$$P=\sum_{k=1}^{3}\sum_{j=1}^{k}\theta_{j}$$

A higher *P* value indicates a higher level of sophistication of the industrial structure.

##### Energy consumption structure

First, the digital economy can stimulate the progress of energy technology, spawn new energy technologies, such as energy storage batteries; new models, such as smart supply chains; new formats, such as new energy efficient vehicles; improve the ECS, directly improve the efficiency of all energy factors, and reduce the intensity of carbon emissions. Alternatively, digital technology, with its advantages of dynamic monitoring, can guide the scheduling of energy between regions (Dhingra et al., 2019), promote the interconnection of clean energy-related fields, optimize the energy structure, promote energy efficiency, and thus, curb the rate of growth of global carbon emissions (Iqbal et al., 2018; Yu et al., 2018). Coal accounts for the largest share in energy consumption in China (Bhattacharya et al., 2015). Therefore, the proportion of coal consumption in energy consumption is used to express the ECS. The total consumption of coal is converted into the total amount of standard coal/energy consumption to indicate the ECS based on previous research (Li et al., 2021).

#### Control variables

Control variables are shown in Table 2.

**TABLE 2:** Control variables.

Control variable	Definition	References
Environmental regulation (*Er*)	It is characterized by the pollution control cost per unit industrial output value, i.e., the ratio of the investment completed in the industrial pollution control project this year to the industrial added value per 1,000 yuan.	Yao et al., 2018
Marketization *(Market)*	It refers to the Report on China’s Marketization Index by province.	Sun and Huang, 2020
Infrastructure (*Infra*)	It is measured by the amount of investment in fixed assets as a percentage of GDP.	Cheng et al., 2019
Population density (*Lnpop*)	It is the logarithm of the ratio of the number of resident people to the geographical area at the end of the year.	Lan et al., 2021
Government intervention (*Gov*)	It is the ratio of local government public finance budget expenditure to regional GDP.	Lan et al., 2021
Openness to the outside world (*Open*)	It is the ratio of total import and export to regional GDP of each region.	Usman et al., 2021

### Data sources and description

Considering the availability of data and the consistency of time ranges, this article studied 30 provinces and municipalities that were directly under the control of Central Government and autonomous regions in China (excluding Tibet, Hong Kong, Macao, and Taiwan) from 2013 to 2018. The data used in the study were derived from the *National Bureau of Statistics, the China Statistical Yearbook*, the *China Environmental Statistics Yearbook*, the *China Energy Statistics Yearbook*, the *China Fixed Asset Investment Statistical Yearbook*, the *National Science and Technology Statistical Yearbook*, and the EPS database, and the missing data were filled by interpolation. Descriptive statistical results of the variables are shown in Table 3.

**TABLE 3:** Results of a descriptive statistical analysis of the variables.

Variable	Obs.	Mean	SD	Min	Max
*lnC*	180	4.2321	0.6933	2.3979	5.9989
*DIGE*	180	0.1722	0.1372	0.0323	0.7182
*Inds*	180	1.2120	0.6501	0.6326	4.3475
*Ecs*	180	0.6631	0.3199	0.0272	1.7307
*Er*	180	0.4269	0.4214	0.0469	3.0984
*Market*	180	6.8899	1.9295	2.5300	10.9000
*Infra*	180	0.8899	0.2982	0.2117	1.5965
*Lnpop*	180	5.4685	1.2939	2.0675	8.2696
*Gov*	180	0.2675	0.1144	0.1237	0.7534
*Open*	180	0.2552	0.2586	0.0123	1.2695

## Empirical results and discussion

### Estimation results of the basic model

According to the basic model (1), linear estimation results of the impact of the digital economy on provincial carbon emission intensity are shown in Table 4. Considering the core explanatory variables, the estimated coefficient of *DIGE* in column (1) was −2.123, and it was highly significant (*p* < 0.01). The coefficient of *DIGE* was still significantly negative in column (2) with the control variables, indicating that even after controlling for other influencing factors, there was still a significant negative influence, which is similar to the findings of Xiao and Jiang (2021). With its data resource sharing and technological innovation spillover effects, the digital economy is a significant promoter of industrial green transformation and changes in the mode of economic development, and the ecological benefits of the digital economy have great potential to achieve the goal of China’s “carbon peaking and carbon neutrality.”

**TABLE 4:** Results of a benchmark regression on the impact of digital economy on the intensity of carbon emissions.

	(2)	(3)
*DIGE*	−2.123***	−1.903***
	(0.227)	(0.187)
*Er*		6.840***
		(2.483)
*Market*		−0.0288*
		(0.0162)
*Infra*		−0.0786*
		(0.0464)
*Lnpop*		1.073**
		(0.476)
*Gov*		−0.202
		(0.345)
*Open*		0.205
		(0.164)
*Constant*	4.598***	−1.068
	(0.0391)	(2.605)
Province FE	YES	YES
Observations	180	180
Number of ID	30	30
*R* ^2^	0.650	0.687

The standard errors are in parentheses. *p < 0.1; **p < 0.05; ***p < 0.01.

In terms of control variables, the coefficient of environmental regulation (*Er*) was significantly positive, indicating that environmental regulation and governance are inefficient, resulting in the phenomenon of a “green paradox” (Okullo et al., 2020). It is necessary to use digital technology to enhance the effect of reducing carbon emissions owing to environmental regulation because resource allocation requires accurate and dynamic feedback. The coefficient of population density (*Lnpop*) was significantly positive, indicating that the intensity of carbon emissions will increase as the population density grows. The primary reason is that while the concentration of population and the use of digital technologies will improve the efficiency of utilization of resources and energy and positively reduce carbon emissions, they have not yet offset the negative effect of carbon intensification caused by the increased demand for domestic and production energy stimulated by the increase in population. Openness to the outside world (*Open*) and government intervention (*Gov*) have yet to pass the significance test, which could be owing to the fact that bringing in foreign capital can easily lead to technological dependency or trigger the “pollution paradise effect” (Wang X. et al., 2022), which is not conducive to domestic technological innovation. In addition, the budgetary expenditures for energy saving and environmental preservation of local governments could be inadequate, resulting in a failure to significantly reduce carbon emissions. The coefficients of marketization (*Market*) and infrastructure (*Infra*) were both negative. This indicates that a higher level of marketization will result in a more inclusive development of digital industries, and market players will increase their investment preferences for human capital and digital frontier technology research and development. In addition, under the guidance of digital power strategy, the government will increase investment in digital infrastructure and digital platform construction, which will support research and development and the application of technologies, such as energy-efficient recycling.

### Estimation results of the mediation effect model

It is necessary to investigate the influencing mechanism and explore the mediation effect of industrial structure advancement and ECS to optimize the reduction of carbon emissions. To verify whether the mechanism is valid, the mediation effect model was used for empirical analysis, and Sobel and Bootstrap tests were performed. The test results are shown in Table 5.

**TABLE 5:** The mediating effect of the digital economy on the intensity of carbon emissions.

	Model 1	Model 2	Model 3	Model 4	Model 5
	*lnC*	*Inds*	*lnC*	*ECS*	*lnC*
*DIGE*	−1.903*** (0.187)	2.337** (0.277)	−1.044*** (0.193)	−0.459*** (0.100)	−1.376*** (0.158)
*Inds*			−0.367*** (0.047)		
*ECS*					1.149*** (0.124)
Control variable	YES	YES	YES	YES	YES
Province FE	YES	YES	YES	YES	YES
*R* ^2^	0.9867	0.9666	0.9905	0.9820	0.9916
Obs.	180	180	180	180	180
Sobel test	*Z* = −5.701, *p*-value = 1.189e−08			*Z* = 2.737, *p*-value = 0.000	
Proportion of total effect that is mediated: 0.4511				0.2771	
Bootstrap test	*Z* = −4.77, *p*-value = 0.000			*Z* = −2.52, *p*-value = 0.012	

The standard errors are in parentheses. **p < 0.05; ***p < 0.01.

According to model (1), the digital economy can significantly promote the reduction of carbon emissions (−1.903). Model (2) verified whether the digital economy improves the advancement of industrial structure. The regression coefficient for this model was significantly positive (2.337) (*p* < 0.05). In model (3), the intermediate variable of industrial structure advancement was substituted into the regression equation, and the coefficient (−1.044) of core explanatory variable of the digital economy decreased compared with model (1), although it was still highly significant (*p* < 0.01). The size of the intermediary effect was 0.8584 = (−1.044) to (−1.903), which accounts for 45.11% of the total effect. This indicates that digital platforms promote the spread of low carbon and environmental protection concepts. The digital economy strengthens market competition, and factors flow to industries with high rates of return and environmental friendliness. Digital technology efficiently integrates and allocates resources to all parts of the supply chain, rendering production intelligent, intensive and flexible, and reducing resource losses and pollution. Thus, the advancement in industrial structure is one of the critical mechanisms of the digital economy to inhibit carbon emissions.

In model (4), the regression coefficient (−0.459) of the impact of the digital economy on the ECS was significantly negative (*p* < 0.01), indicating that the digital economy cannot improve the ECS. According to model (5), the size of the intermediary effect was 0.5272 = (−1.376) to (1.903), which accounted for 27.71% of the total effect. The indicates that digital finance alleviates the financing difficulties that could be encountered by innovation in green technology by the energy industry. Digital technology provides strong support for the construction of energy and environmental monitoring platforms, optimizes the extraction of energy, production and transportation, explores the construction of distributed and clean energy systems, reduces the use of traditional fossil energy, such as coal in energy consumption, optimizes the energy structure, and improves the efficiency of energy use. Thus, the ECS is also an important mechanism.

### Estimation results of the Hansen threshold effect

The analysis above shows that the *DIGE* has different development stages. Therefore, it is necessary to explore its non-linearity and the possible phased impact. Quantifying the threshold effect is conducive to formulating corresponding feasible policies.

#### Test of the existence of the threshold effect

The results of a test of the existence of the threshold effect of digital economy is shown in Table 6.

**TABLE 6:** Test of the existence of the threshold effect.

	Number of thresholds	*F*-value	*p*-Value	Critical value			Sampling times
				10%	5%	1%	
Digital economy development	Single threshold	27.67*	0.0640	24.1852	29.0407	37.3542	1,000
	Double thresholds	27.69**	0.0260	18.7808	23.3614	34.2829	1,000
	Three thresholds	19.34	0.7020	58.0924	64.4716	84.3550	1,000

Sampling times refer to the repeated sampling times through bootstrap. *p < 0.1; **p < 0.05.

According to the significance test results in Table 6, double threshold, compared with single threshold, which is more detailed, was chosen to analyze the nonlinear characteristics.

### Threshold effect analysis

The threshold regression results are shown in Table 7.

**TABLE 7:** Threshold regression results.

	DIGE			Er	Market	Infra	Lnpop	Gov	Open	Constant
	DIGE ≤ 0.0508	0.0508 < DIGE ≤ 0.0525	DIGE > 0.0525							
Coefficient	−0.606	4.254***	1.874***	0.062***	–0.0213	–0.0316	1.229***	−0.559*	0.230	–1.936
T value	−0.913	4.685	–11.497	2.847	–1.500	–0.771	2.998	–1.833	1.608	0.854

*p < 0.1; ***p < 0.01.

As shown in Table 7, two obvious thresholds – 0.0508 and 0.0525 – existed during the process of promotion of the reduction in carbon emissions by the digital economy. The findings reveal an “inverted N-shaped” curve link between the development of regional digital economies and carbon emissions. These results are different from previous research, which identified an inverted U-shaped relationship (Li et al., 2021). There are three situations.

(1) When the digital economy development index (DIGE) < 0.0508, the impact of the digital economy development on carbon emissions was negative but not significant, thus, indicating that the small-scale application of digital technology in the “nascent” stage of the digital economy had overturned the operation mode of some industries (Liu et al., 2020). This resulted in better resource allocation, lowered production and transaction costs, and an improvement in the productivity of traditional industries. This initially shows the effect of green technology innovation and reduces carbon emissions to some extent.
(2) When 0.0508 < DIGE < 0.0525, the regression coefficient of the digital economy was significantly positive, indicating that at this stage, the digital economy failed to effectively curb carbon emissions, and its development had reached a “bottleneck.” The possible reason is that the government may have boosted its investment in the construction of digital economy facilities, motivated by both the economic and environmental benefits at the start-up stage of *DIGE*. However, the construction cycle for the digital economy is lengthy and costly. On the one hand, data sharing and circulation encounter numerous challenges and eke out environmental protection expenditures. Alternatively, the design, installation, and operation of digital infrastructure consumes significant amounts of energy (Berkhout and Hertin, 2004; Hilty et al., 2006). Furthermore, some extremely polluting and energy-consuming projects are being promoted under the pretense of new infrastructure, which has a pernicious influence on the reduction of carbon emissions.
(3) When the DIGE > 0.0525, the digital economy coefficient was significantly negative (*p* < 0.05), indicating that the digital economy has crossed the second threshold and entered the “mature stage” in which the DIGE has an “enabling effect” on improving the reduction in efficiency of reducing carbon emissions. The primary reason is that the digital infrastructure has improved further as a result of the implementation of relevant government support policies and the continual advancement in information and communication technology. Therefore, the digital economy plays a more effective role in the industrial structure advancement, thus, accelerating the low-carbon transformation of energy demand structure (Ren et al., 2021). In addition, digital technology facilitates the disclosure of climate-related data, such as carbon emissions, and makes it feasible to lock carbon emission sources, detect carbon emissions, and measure other environmental indicators, enabling the formation of a national unified carbon emissions trading market.

### Estimation results of the spatial spillover effects

Carbon dioxide is a major source of the greenhouse effect, which is coupled with the increasing geographic proximity to economic exchanges (Zhao et al., 2022). Therefore, carbon emissions as an unintended output of economic activity may have spatial spillovers.

#### Test of existence of spatial effects

At first, the carbon emissions were tested for spatial effects. Based on the adjacency and the geographic distance matrices, the Moran index of carbon emissions from 2013 to 2018 (Table 8) was calculated, and the results show that the global Moran index was significantly positive, indicating that the spatial distribution of carbon emissions in 2013–2018 has obvious spatial dependence – “high-high” and “low-low” aggregation phenomena.

**TABLE 8:** Moran’s I test of the intensity of carbon emissions from 2013 to 2018.

*lnC*	Spatial adjacency matrix		Geographic distance matrix	
Year	Moran’s I	*Z*-value	Moran’s I	*Z*-value
2013	0.195**	1.905	0.072***	3.352
2014	0.208**	2.011	0.070***	3.269
2015	0.204**	1.982	0.062***	3.039
2016	0.215**	2.069	0.065***	3.087
2017	0.203**	1.987	0.054***	2.876
2018	0.202**	1.976	0.056***	2.923

**p < 0.05; ***p < 0.01.

#### Spatial model selection and regression result

After the LM, LR, and Wald tests were performed sequentially, the spatial Durbin model with fixed effects was selected to test the spatial effect. To further explore its spatial effect, the results of spatial Durbin model (*SDM*) and spatial autoregressive (SAR) model were compared in Table 9.

**TABLE 9:** Estimation results of the spatial effect.

Model setting	SDM		SAR	
Spatial matrix	Spatial adjacency matrix	Geographic distance matrix	Spatial adjacency matrix	Geographic distance matrix
ρ	0.563***	0.347*	0.694***	0.794***
	(0.0738)	(0.200)	(0.0494)	(0.0522)
*DIGE*	−0.724***	−0.637***	−0.953***	−0.792***
	(0.172)	(0.175)	(0.133)	(0.138)
*W* × *DIGE*	−0.253	−0.296		
	(0.255)	(0.541)		
Control variables	YES	YES	YES	YES
Direct effect	−0.835***	−0.642***	−1.129***	−0.885***
	(0.180)	(0.181)	(0.146)	(0.144)
Indirect effect	−1.339***	−0.761	−1.957***	−3.170***
	(0.408)	(0.730)	(0.415)	(1.035)
Total effect	−2.174***	−1.402*	−3.086***	−4.055***
	(0.475)	(0.760)	(0.487)	(1.078)
Obs.	180	180	180	180
*R* ^2^	0.334	0.355	0.179	0.366
Log-likelihood	281.605	292.8246	274.4410	277.6310
Province FE	YES	YES	YES	YES

Standard errors in parentheses. DIGE, digital economy development index; FE, fixed effect; SAR, spatial autoregressive; SDM, spatial Durbin model. ***p < 0.01; *p < 0.1.

From an overall point of view, every coefficient of *DIGE* (−0.724, −0.637, −0.953, and −0.792) in Table 9 was significantly negative, indicating that the DIGE can effectively inhibit carbon emissions. The spatial lag term in both models was significantly positive at least to *p* < 0.1, indicating that there are obvious characteristics of inter-regional interaction in the carbon emissions. This could possibly be owing to the similarity of the factor allocation, energy utilization, industrial development, and production methods between neighboring provinces, resulting in a spatial correlation between the carbon emissions.

Direct and indirect effects need to be measured by a partial differential interpretation of variable variations to prevent systemic bias. Since simple point regression results cannot accurately estimate the spatial spillover effect between regions, the coefficient of the spatial interaction term cannot directly reflect the marginal impact. From the perspective of direct effect, the DIGE can significantly suppress local carbon emissions. The local digital economy development will reduce local carbon emissions by 0.835 units for every unit increased. For indirect effects, under the adjacency weight matrix, the digital economy and carbon emission intensity of neighboring regions significantly negatively correlate, and for every unit increase in DIGE in the local area, the intensity of carbon emissions in the neighboring regions will be reduced by 1.339 units.

It is worth noting that the indirect effect was larger than the direct effect, which indicates that the positive promotion effect of the local development of the digital economy on the efficiency of the reduction in carbon emissions of neighboring regions (the “neighbor effect”) is greater than the negative inhibitory effect (“local effect”) of local carbon emissions. There are several reasons for this. First, the DIGE in neighboring areas promotes the formation of high-tech as the leading industry, digital manufacturing as the support industry, and the vigorous development of the tertiary industry industrial pattern (Wang et al., 2021). Secondly, the advancement of industrial structure, diffusion of innovative green technology, improvement in the efficiency of energy utilization, and the inhibition of radiation of carbon emissions to surrounding areas through the mechanism of industrial chain transmission. Third, a higher degree of digitization increasingly attracts the influx of labor, resources, and other elements in neighboring areas, resulting in a “siphon effect.” This effect will intensify the demand for energy consumption, increase the cost of energy conservation and reduce emissions in neighboring areas. Fourth, owing to the inhibition of “offset effect,” the local region will surpass neighboring areas in information technology and form a coordinated low-carbon economic growth model. This results in a downward trend in the intensity of energy consumption, and therefore, there is a large “neighbor effect” on carbon emissions.

## The robustness test

### Temporal lags test

Owing to the possibility of bias on the empirical results by omitted variables and two-way causality, this study lagged the core explanatory variables by one and two periods for the baseline regression test. The regression results of the digital economy (−2.119 and −1.810) were significantly negative in columns 2 and 3 of Table 10, which verified the results.

**TABLE 10:** Estimation results of the robustness test.

Model setting	Baseline regression		SDM	
	Lag one period	Lag two periods	Economic distance matrix	Economic geography nested matrix
*DIGE*	−2.119***	−1.810***	−0.451**	−0.637***
	0.228	0.212	(0.175)	(0.175)
*W* × *DIGE*			−0.470	−0.296
			(0.320)	(0.541)
ρ			0.500***	0.347*
			(0.097)	(0.200)
Control variables	YES	YES	YES	YES
Direct effect			−0.513***	−0.798***
			(0.187)	(0.195)
Indirect effect			−1.321**	−2.216***
			(0.554)	(0.560)
Obs.	150	120	180	180
*R* ^2^	0.6234	0.5341	0.253	0.328
Log-likelihood			288.510	285.762
Province FE	YES	YES	YES	YES

The standard errors are in parentheses. DIGE, digital economy development index; FE, fixed effect; SDM, spatial Durbin model. *p < 0.1; **p < 0.05; ***p < 0.01.

### Replace the weight matrix

We explored the spatial effect based on the adjacency and geographical distance matrices. Because the economic development of various regions cannot be viewed in isolation, in addition to geographical attributes, economic characteristics are also the causes of the spatial spillover of carbon emissions. Therefore, economic distance and economic geography nested matrices were introduced to investigate the robustness of the spatial measurement model.

The results of Table 10 show that under the two weight matrices, the spatial lag term coefficient of carbon emissions was still significantly positive factoring in economic factors, and the spatial Durbin panel model effect decomposition only changed slightly, but the significance of the variables did not change. Therefore, it can be concluded that the digital economy development can improve the efficiency of local and neighboring areas to robustly and credibly to reduce carbon emissions.

## Further analysis-regional heterogeneity test

The empirical results described above show that the DIGE has a strong spatial spillover effect on carbon emissions in general. However, there could be regional heterogeneity in which the local spatial correlations are different or even contradictory considering that resource endowments, economic foundation and macro development strategies are heterogeneous in different regions. Based on the criteria of the National Development and Reform Commission and the National Geographic Region Division, this study analyzed data from the eastern, central, western, coastal, and inland aspects, respectively. A more realistic economic geography matrix was selected as the spatial weight matrix to factor in space limitations. The results are shown in Table 11.

**TABLE 11:** Estimation results in different regions of China.

	Eastern China	Central China	Western China	Coastal areas	Inland areas
*DIGE*	−1.091***	−0.536**	−0.313	−0.709**	−1.466***
	(0.262)	(0.211)	(0.302)	(0.327)	(0.156)
*Er*	0.0167	−0.0176	−0.0424**	0.0521	−0.0133
	(0.0372)	(0.0388)	(0.0176)	(0.0340)	(0.0167)
*Market*	−1.547***	0.0279	0.105	−0.928	0.258
	(0.515)	(0.421)	(0.301)	(0.565)	(0.258)
*Infra*	2.600***	−3.700***	1.243**	3.265***	1.097***
	(0.673)	(0.744)	(0.586)	(0.740)	(0.335)
*Lnpop*	−0.0528	−0.124***	−0.151***	−0.126*	−0.181***
	(0.0625)	(0.0278)	(0.0438)	(0.0647)	(0.0339)
*Gov*	0.0218	0.00747	0.0246	−0.0146	0.0575***
	(0.0170)	(0.0148)	(0.0171)	(0.0152)	(0.0131)
*Open*	0.198	−0.163	0.194	0.285**	0.742***
	(0.139)	(0.347)	(0.230)	(0.139)	(0.195)
Direct effect	−1.041***	−0.542**	−0.299	−0.401	−1.461***
	(0.249)	(0.213)	(0.316)	(0.355)	(0.162)
Indirect effect	−1.679***	−0.669*	0.0166	−1.591***	0.277
	(0.498)	(0.345)	(0.772)	(0.466)	(0.398)
Total effect	−2.720***	−1.212***	−0.282	−1.992***	−1.185***
	(0.538)	(0.343)	(0.812)	(0.441)	(0.392)
Obs.	66	48	66	66	114
*R* ^2^	0.282	0.440	0.174	0.258	0.236
Log-likelihood	315.701	285.760	237.990	222.386	277.515
Province FE	YES	YES	YES	YES	YES

The standard errors are in parentheses. DIGE, digital economy development index; Er, environmental regulation; FE, fixed effect; Gov, government intervention; Infra, infrastructure; Lnpop, population density; Open, openness to the outside world. *p < 0.1; **p < 0.05; ***p < 0.01.

As shown in Table 11, the impact of the DIGE on carbon emission intensity has obvious spatial differentiation in the three major regions, as well as coastal and inland areas, which reflects the current situation of unbalanced development in China. Considering the regression coefficient of *DIGE* and the decomposition of spatial spillover effects, there are two interesting results. (1) Compared with the western region, the DIGE has significant “neighbor” and “local” effects on the reduction of carbon emissions in the central and eastern regions. First, there is a large gap between the construction of digital infrastructure, and digital technology penetration in the western region is low, thus, failing to exert the digital economy’s green innovation technology effect. Secondly, the western areas are mostly resource-dependent provinces with high levels of carbon emissions, and the shift of polluting firms from the central and eastern regions to the western regions increases the strain on environmental management in the western regions. (2) The DIGE in coastal provinces and cities can significantly reduce carbon emissions in neighboring areas compared with the positive and insignificant indirect spillover effects in the inland. Therefore, it is more important to focus on the efforts to reduce carbon emissions in inland areas because the overall development of economic and ecological environmental benefits can only be promoted through collaborative prevention and control of the coastal and inland areas.

## Conclusion and policy implications

Achieving “Dual Carbon” goals and low-carbon development is a long-term complex process, and the digital economy influences the levels of carbon emission from multiple aspects and dimensions. Based on the panel data from 2013 to 2018, this study empirically examined the non-linear and spatial impact of the digital economy on carbon emissions and its inherent mechanism. The following conclusions were drawn. First, the development of China’s digital economy can effectively promote the reduction of carbon emissions and has a significant effect of spatial spillover in general. Secondly, the threshold regression shows an “inverted N-type” relationship between them. In particular, the DIGE inhibits carbon emissions during the embryonic and mature stages and has a negative impact on the reduction of emissions during its bottleneck period. Third, there is obvious regional heterogeneity. The DIGE in central and eastern regions has significant “local” and “neighboring” effects on carbon emission reduction compared with the western region. The inland “neighbor” effect is stronger than in coastal areas. Fourth, the DIGE can indirectly promote improvement in the efficiency of reducing carbon emissions through the advancement of industrial structure and the optimization of ECS. Based on the research conclusions of this article, the following policy recommendations are proposed:

(1) The layout of digital infrastructure and basic technologies, such as blockchain and the internet, should be appropriately accelerated. Investment in the research and development of key technologies in the digital field should be increased, and the support of digital technologies for carbon reduction should be enhanced. First, it is necessary to accelerate industrial digitalization, apply green-oriented digital technologies to all aspects of the traditional supply chain, reduce resource misallocation and loss rates, and improve the green total factor productivity. Secondly, digital industrialization should be promoted, and the research and development of energy-saving and emission-reduction technologies, such as carbon capture and carbon storage, should be accelerated.
(2) Each region should fully consider regional heterogeneity and grasp the stage of its own digital economy development. It should establish a digital economy development system with local characteristics based on local advantages. In particular, the central government should conduct appropriate policies to support technologies and raise funds for digital construction in the western region. The digital economic exchanges and interactions between regions should be improved considering the spatial spillover effect, and the inland regions should learn from the green development model of the digital economy in the coastal regions, break the geographical barriers of new models and new formats of the digital economy, gradually narrow the difference between regional digital innovation and application capabilities, and achieve a win-win situation between economic development and carbon reduction.
(3) It is necessary to exert the effect of advancement of industrial structure and the optimization of energy consumption during the process. First, the digital economy can drive industrial advancement from many aspects, such as by promoting production efficiency, industrial integration and efficiency in the innovation of green technology, and guiding green consumption concepts. Alternatively, digital technology can monitor the total amount of energy in real time, accurately dispatch and distribute energy, alleviate the pressure of traditional energy peak regulation, motivate the development of new energy, optimize energy operation and management, greatly improve energy utility efficiency, and reduce the consumption of traditional fossil energy. This indirectly enhances the effect of digital technology at reducing carbon emissions.
(4) The government’s overall planning, regulation and governance capabilities in digitalization should be strengthened to ensure the healthy development of DIGE, protect the vitality of various innovative entities in the market, and severely punish digital oligarchs whose monopoly of data resources causes the threshold rise of the digital industry. Simultaneously, university research institutions should be encouraged to open cutting-edge courses in the digital field to improve the digital capabilities of students, while increasing subsidies for entrepreneurs, cultivating digital innovative talents, narrowing the “digital divide,” and accumulating human capital for the sustainable positive impact of the digital economy on carbon emission reduction.

This study provides important evidence on the relationship between the digital economy and carbon emissions at the provincial, municipal and autonomous levels. However, there are still some limitations. In particular, this study focused on the static relationship between the digital economy and carbon emissions, but it ignored whether there is an interesting link between the two in time and whether there are other transmission mechanisms. In the future, with the increasing penetration of the digital economy and the continuous promotion of China’s 2060 carbon neutrality target, systematic GMM, dynamic thresholds, dynamic spatial Durbin models and moderating effects can be used to explore the dynamic relationship and pathways. In addition, future research could be directed down to the city and county levels.

## Data availability statement

The original contributions presented in the study are included in the article/supplementary material, further inquiries can be directed to the corresponding authors.

## Ethics statement

This study was reviewed and approved by the local ethics research committee of Xinjiang University. Written informed consent was obtained from all participants for their participation in this study.

## Author contributions

XH and SW: writing the original draft. YL: conceptualization, methodology, data curation, and supervision. YPX: review and editing. YX: topic selection and writing. All authors contributed to the article and approved the submitted version.
